# Diabetes and COVID-19

**DOI:** 10.1515/biol-2021-0034

**Published:** 2021-03-25

**Authors:** Zohair Jamil Gazzaz

**Affiliations:** Department of Internal Medicine, Faculty of Medicine, Rabigh, King Abdulaziz University, Jeddah 21577, Saudi Arabia

**Keywords:** diabetes mellitus, COVID-19, ACE2, cytokine storm, CVD

## Abstract

Several factors are linked with a higher risk of mortality from Coronavirus disease-19 (COVID-19), including male gender, increased age, hypertension, diabetes mellitus, obesity, cardiovascular diseases, chronic obstructive pulmonary disease, and cancer. Hyperglycemic COVID-19 patients have severe clinical problems, increased ICU admittance, machine-driven ventilation, and a substantial rise in inflammatory markers. Among all patients, those with diabetes or hyperglycemia have a two- to four-fold increase in mortality and severity of COVID-19 than those without diabetes. The primary cause of mortality in COVID-19 patients with diabetes is compromised immune response to viral infections. Increased blood sugar level probably affects intracellular degradation of bacteria, neutrophil chemotaxis, and phagocytosis, thus improving viral binding affinity and entry and decreasing virus clearance. In addition, it has significant effects on the proteins by inducing glycosylation and altering the composition of complements, and glycosylation renders cells susceptible to viral inflammation and damage. The treatment of COVID-19 in patients with diabetes requires an integrated team approach to minimize the risk of medical complications and mortality. Moreover, physicians should adopt proactive strategies to care for persons with comorbidities. This strategy would help reduce the frequency of complications and mortality among patients and load on the healthcare system.

## 1 Introduction

The COVID-19 pandemic has profoundly influenced the economic outlook and psychosocial well-being worldwide. The global economy and tourism are predicted to struggle a lot in the years to come. Millions have been unemployed, industries are down, and virtually every part of life has been affected [1].

Wuhan City, Hubei Province, China was the starting point of this viral pandemic [2]. Severe Acute Respiratory Syndrome Coronavirus-2 (SARS-CoV-2) is a part of the Coronaviridae family that has also previously caused the SARS epidemic in 2002 and MERS epidemic in 2008, killing 774 and 858 people, respectively [2,3].

The Coronaviridae is a family of enveloped viruses with a positive-sense single-stranded RNA genome [4,5]. SARS-CoV-2 is structurally different from other viruses, consisting transmembrane crown-like glycoprotein spikes [6]. The virus reaches the host cells using the angiotensin-converting enzyme 2 (ACE2) receptor [7]. Once inside, the virus captures the host cell’s genetic system to make viral proteins and their genetic material. After replication is complete, the virus leaves the cell through exocytosis; the stress of the virus triggers apoptosis of the cell [8]. The apoptosis or necrosis of the virally infected cells triggers an inflammatory response via the activation of proinflammatory cytokines. These high amounts of cytokines eventually lead to a “cytokine storm” in the body [9]. The cytokine storm causes multiple organ failure and hyper-inflammation [10,11].

A recent study from UAE reported 10% mortality among COVID-19 patients with diabetes [12]. For newly diagnosed diabetic cases, mortality and mechanical ventilation needs were considerably higher relative to people with pre-existing diabetes. Patients with COVID-19 infection are negatively impacted by uncontrolled hyperglycemia. A potential risk of complications is recently diagnosed and previously undiagnosed hyperglycemia [12].

Glycemia adjustment in hospitalized patients is vital in the context of the COVID-19 pandemic, and screening to identify undiagnosed cases of diabetes is markedly important [12]. A meta-analysis reported a 9.7% prevalence of diabetes mellitus (DM) in COVID-19 patients in China [13], which is comparable to the DM prevalence in China. A UK study described hospital death of 23,804 COVID-19 patients, of which 32% had type 2 DM and 1.5% had type 1 DM, with 2.03- and 3.5-fold risk of death in hospital relative to non-DM patients, respectively [14]. A retrospective Chinese study suggested that COVID-19 patients with diabetes had a higher incidence of hypertension (56.9%), cardiovascular disease (CVD) (20.9%), and cerebrovascular disease (7.8%) compared to non-DM patients (28.8, 11.1, and 1.3%, respectively) [15]. A population-based cohort study from England reported a greater possibility of death from COVID-19 in patients with diabetes (either type 1 or type 2 DM) with HbA 1c > 10% relative to those with HbA 1c < 6.5% [16].

## 2 COVID-19 and comorbidities

The dynamics of pathophysiology are still being recognized around the world. Elderly COVID-19 patients with comorbidities have severe and fatal issues [17,18]. A nationwide analysis conducted on 1,590 laboratory-confirmed patients in China had a mean age of 49 years, with 399 (25.1%) patients had at least one comorbid disease. Hypertension was the most predominant (16.9%), followed by DM (8.2%). A small number of patients, 130 (8.2%), had two or more comorbidities. The findings suggest that COVID-19 patients with some comorbidity had worse clinical results compared to those without an associated condition, and more comorbidities were also associated with poorer clinical outcomes [19]. In another case series that had 5,700 confirmed cases of COVID-19, the commonly prevalent comorbidities were hypertension (3,026; 56.6%), obesity (1,737; 41.7%), and DM (1,808; 33.8%), who were hospitalized in the New York City region [20].

One Chinese meta-analysis involved 1,527 patients and considered hypertension to be the most frequent cardiovascular comorbidity in COVID-19 (17.7%), cardio-cerebrovascular disease (16.4%), and DM (9.7%). Furthermore, diabetic or hypertensive patients had a two-fold increased risk of developing serious complications or requiring ICU admissions. In contrast, cardio-cerebrovascular illness patients had a three-fold rise [13].

## 3 How COVID-19 impacts DM patients

DM is a metabolic disease illustrated by chronic high circulating blood glucose concentration in the body [21]. The worldwide prevalence of DM has increased drastically, with 463 million people suffering from the disease globally in 2019, with 95% of type 2 DM [21]. Currently, many patients who have diabetes remain undiagnosed with the disease. The therapeutic intervention has reduced disease complications, making it necessary to detect diabetes early on in its course. DM occurs throughout the world, but it is found explicitly in the more developed countries, where the majority of the patients are in the age group between 45 and 64 years. A relationship has been clinically recognized between diabetic individuals and infections [22]. A virus that causes pneumonia, particularly influenza, is prevalent and presents a much more profound threat in older patients with type 2 DM [23,24].

There is a higher chance of poor prognosis and death for COVID-19 patients with diabetes. With the high global incidence of diabetes, these individuals constitute a significant portion of the population susceptible to COVID-19. Several factors are linked with a higher risk of mortality from COVID-19, including male gender, increased age, hypertension, DM, obesity, CVDs, chronic obstructive pulmonary disease, and cancer [25].

Reports from countries such as China [13,17,19], Italy [26], and the City of New York [20] have shown that older age and chronic diseases such as DM, hypertension, and extreme obesity are now considered to augment morbidity and death in COVID-19 patients. It is indistinct whether DM leads individually to elevated risk, but blood glucose and DM predict morbidity and death in SARS patients [27].

An American study reported that in 1,122 patients admitted in hospital for COVID-19, the rate of mortality during hospital stays of those suffering from diabetes or hyperglycemia was four-fold higher (28.8%) than that of patients with normal glucose level (6.2%) [28]. A reduced chance of complications and the all-cause death rate were also linked with good glycemic regulation [29]. Al Hayek et al. investigated the factors that may raise the risk of hospital admission of COVID-19 patients with diabetes. They reported patients’ hospitalization was independently associated with a high HbA1c level in Saudi patients [30].

A Chinese meta-analysis reported that high HbA1c is a predictor of the in-hospital mortality of COVID-19 patients [31]. Individuals with elevated HbA1c are at high risk for COVID-19. They should comply with the doctor’s recommendation and precisely track and regulate glucose metabolism regularly [31]. It has been proposed that diabetes could potentiate the severity of SARS-CoV-2 infection via diverse routes that eventually contribute to advanced glycation end products, glucose toxicity, endotheliitis, vital organ injury, and fatality [32].

It is reported that ACE2 receptors mediate the SARS-CoV-2 effects in the host cells. ACE2 receptors are present in the respiratory system, type 1 and 2 alveolar cells of the lungs, hearts, kidney tubules, enterocytes, and pancreas. Studies conducted on rodent models of DM have demonstrated that there has been an augmented manifestation of ACE2 receptors in the lungs, heart, kidney, and pancreas [33,34]. Furthermore, a randomized study found that increased lung ACE2 expression was linked with DM [35]. Furthermore, DM patients have higher circulating Furin levels, a cellular protease that cleaves S1 and S2 domains of spike protein, facilitating the access of SARS-CoV-2 [36]. These studies support that DM patients present a higher COVID-19 risk, and diabetic individuals infected with the disease have impaired virus clearance [37]. ACE2 helps in the catalytic conversion of angiotensin 2 to angiotensin 1–7 or angiotensin 1–9. This conversion is essential as it has a protective effect on the lungs against ARDS [38]. This protective action is because of the antioxidant and anti-inflammatory action of angiotensin 1–7 and angiotensin 1–9. Unfortunately, once the SARS-CoV-2 virus binds with ACE2, it is degraded, preventing any protective action toward the lung parenchyma. This allows angiotensin 2 to cause lung injury [39].

Furthermore, there is more potassium loss in the urine and increased aldosterone secretion by the virus [17]. Nevertheless, more studies need to be conducted to assess the full potential effects of ACE2 expression concerning diabetes and COVID-19. Predictions and decisions can be made if angiotensin receptor blockers, angiotensin-converting enzyme inhibitors, TZDs, GLP-1 agonists, and statins reduce ACE2 appearance in COVID patients.

DM also causes impairment in the activation of the adaptive immune response by inhibiting stimulation of Th1 cell-mediated immunity, thereby leading to a delayed hyperinflammatory reaction in diabetic patients [40]. A study that examined DM effects in mouse models of MERS-Cov infectivity showed that the infection was further prolonged and severe in diabetic male mice. This was because of the variations in CD4+ T cell counts and aberrant cytokine responses [41]. This finding is fairly consistent with SARS-CoV-2 patients, with peripheral counts of CD4+ and CD8T cell counts being abnormally low but subsequently having higher levels of proinflammatory Th17 cells and elevated cytokine count [42,43]. This demonstrated that DM patients have a severely blunted anti-viral response compared to a fairly accentuated inflammatory response because of delayed activation of Th1/Th17 [42]. The SARS-CoV-2 productively infects β-cell and can trigger acute insulin secretion impairment or β-cells loss, which can initiate diabetes [44].

To understand the possible causal association between chronic untreated hyperglycemia and higher death rates in COVID-19 patients, certain biological pathways have been suggested. The primary cause of mortality in COVID-19 patients with diabetes is an inadequate immune response to viral infections [45]. The increased blood sugar level is expected to substantially affect the intracellular degradation of bacteria, neutrophil chemotaxis, and phagocytosis, thus improving viral binding affinity and entry and decreasing virus clearance [46]. In addition, it has significant effects on the proteins by inducing glycosylation and altering the composition of complements [47,48], and glycosylation renders cells susceptible to viral inflammation and damage [49,50]. In addition, endotheliitis may be a potential pathway that triggers organ dysfunction that causes essential COVID-19 disease, aggravated by endothelial dysfunction coupled with chronic hyperglycemia [51].

Multiple variables are associating diabetes with infection severity. The acute inflammatory response can be triggered, intensified, or prolonged by hyperglycemia [52]. It also induces a coagulation and fibrinolysis mismatch, resulting in elevated coagulation factors and relative fibrinolytic system inhibition, fostering a pro-coagulant state [53]. Moreover, SARS-CoV-2 is suspected to be using the ACE2 as the entry receptors on the Langerhans islets. This can lead to moderate to dawning of these cells, leading to moderate hyperglycemia to life-endangering diabetic ketoacidosis [50].

Diabetic people’s vulnerability during a public health epidemic turns out to be evident in the COVID-19 pandemic because of their at least twice higher risk of severe illness or death, particularly in people with poorly regulated diabetes and comorbidities, or both. The pressure on healthcare organizations and the world economy because of COVID-19, compounded by chronic diseases like diabetes, has been enormous [21] (Table 1).

**Table 1:** Outcome risks among COVID-19 patients with diabetes

Authors	Article type	Study population	Prevalence of diabetes	Outcome	Risk
Parohan et al. [25]	Meta-analysis	27,352	—	Mortality	2.41 (1.05–5.51)
Zhu et al. [31]	Meta-analysis	1,180	—	Mortality	2.3 (1.679–3.150)
Kumar et al. [55]	Meta-analysis	16,003	9.8%	Mortality	1.9 (1.37–2.64)
Wei et al. [56]	Prospective	167	6.59%	Severity	10.12 (2.742–37.347)
Simonnet et al. [57]	Retrospective	124	23%	Invasive mechanical ventilation	2.45 (0.67–3.49)
Wu et al. [58]	Retrospective	201	10.9	ARDS	2.34 (1.35–4.05)
Wu et al. [58]	Retrospective	201	10.9%	Mortality	1.58 (0.80–3.13)
Mo et al. [59]	Retrospective	155	9.7%	Refractory COVID-19	2.138 (0.483–9.471)
Huang et al. [60]	Meta-analysis	6,452	26.48%	Mortality	2.12 (1.44–3.11)
Huang et al. [60]	Meta-analysis	6,452	26.48%	ARDS	4.64 (1.86–11.58)
Huang et al. [60]	Meta-analysis	6,452	26.48%	Severe COVID-19	2.45 (1.79–3.35)
Tian et al. [61]	Meta-analysis	4,659	23.8%	Mortality	2.0 (1.7–2.3)

ARDS = acute respiratory distress syndrome.

An extensive survey showed that respondents had a low knowledge score regarding COVID-19. In this respect, it becomes more important to be aware of COVID-19 and its consequences on human health [54]. Establishing a valid association between DM and COVID-19 is imperative in treating DM patients. It would also help in DM care in the present conditions and assist in similar outbreaks in the future. It is also essential to take care of the comorbidities of DM patients while treating DM. Now that the COVID-19 vaccine is available, we need to get vaccinated to save ourselves from this highly infectious disease and save other society members.

The community must also implement social distancing guidelines and protocols to protect those who are more susceptible to morbidity from this disease, such as comorbid individuals or older aged people. Comorbid individuals are those who are more severely being infected by the virus. Patients with chronic illnesses like diabetes, hypertension, and respiratory diseases are more threatened by the virus than those without any underlying conditions.

The treatment of COVID-19 patients with diabetes requires an integrated team approach to minimize the risk of medical complications and mortality [44]. Besides, doctors should incorporate pragmatic measures to treat patients with comorbidities. This strategy would help reduce the frequency of complications, mortality among patients, and the healthcare system’s overall load.

## 4 Conclusion

COVID-19 is an ongoing pandemic with new information concerning the disease continually emerging. Diabetes is an essential comorbid factor for COVID-19, and diabetic patients must take the necessary steps to prevent them from becoming infected. More research is required to cater a clearer understanding of the prevalence of morbidity, mortality, and pathophysiology of COVID-19 in diabetic patients.

## References

[1] Ahmad T, Haroon, Baig M, Hui J. Coronavirus disease 2019 (COVID-19) pandemic and economic impact. Pak J Med Sci. 2020;36(COVID19-S4):S1–6. 10.12669/pjms.36.COVID19-S4.2638 PMC7306969 32582318

[2] Huang YT, Lee YC, Hsiao CJ. Hospitalization for ambulatory-care-sensitive conditions in Taiwan following the SARS outbreak: a population-based interrupted time series study. J Formos Med Assoc. 2009;108:386–94. 10.1016/S0929-6646(09)60082-6 PMC7135451 19443292

[3] Chan‐Yeung M, Xu RH. SARS: epidemiology. Respirology. 2003 Nov;8:S9–14. 10.1046/j.1440-1843.2003.00518.x PMC7169193 15018127

[4] Cui J, Li F, Shi ZL. Origin and evolution of pathogenic coronaviruses. Nat Rev Microbiol. 2019;17:181–92. 10.1038/s41579-018-0118-9 PMC7097006 30531947

[5] Su S, Wong G, Shi W, Liu J, Lai AC, Zhou J, et al. Epidemiology, genetic recombination, and pathogenesis of coronaviruses. Trends Microbiol. 2016;24:490–502. 10.1016/j.tim.2016.03.003 PMC7125511 27012512

[6] Xu X, Chen P, Wang J, Feng J, Zhou H, Li X, et al. Evolution of the novel Coronavirus from the ongoing Wuhan outbreak and modeling of its spike protein for risk of human transmission. Sci China Life Sci. 2020;63:457–60. 10.1007/s11427-020-1637-5 PMC7089049 32009228

[7] Harmer D, Gilbert M, Borman R, Clark KL. Quantitative mRNA expression profiling of ACE 2, a novel homologue of angiotensin converting enzyme. FEBS Lett. 2002;532:107–10. 10.1016/s0014-5793(02)03640-2 12459472

[8] Li YC, Bai WZ, Hashikawa T. Response to commentary on the neuroinvasive potential of SARS‐CoV‐2 may play a role in the respiratory failure of COVID‐19 patients. J Med Virol. 2020;92:707–9. 10.1002/jmv.25824 PMC7228344 32246783

[9] Palm NW, Medzhitov R. Not so fast: adaptive suppression of innate immunity. Nat Med. 2007;13:1142–4. 10.1038/nm1007-1142b PMC7095835 17917657

[10] Mehta P, McAuley DF, Brown M, Sanchez E, Tattersall RS, Manson JJ. Across speciality collaboration. COVID-19: consider cytokine storm syndromes and immunosuppression. Lancet. 2020;395:1033. 10.1016/S0140-6736(20)30628-0 PMC7270045 32192578

[11] Wan S, Yi Q, Fan S, Lv J, Zhang X, Guo L, et al. Characteristics of lymphocyte subsets and cytokines in peripheral blood of 123 hospitalized patients with 2019 novel coronavirus pneumonia (NCP). MedRxiv. 2020.

[12] Hafidh K, Abbas S, Khan A, Kazmi T, Nazir Z, Aldaham T. The clinical characteristics and outcomes of COVID-19 infections in patients with diabetes at a tertiary care center in the UAE. Dubai Diabetes Endocrinol J. 2021;26:1–6.

[13] Li B, Yang J, Zhao F, Zhi L, Wang X, Liu L, et al. Prevalence and impact of cardiovascular metabolic diseases on COVID-19 in China. Clin Res Cardiol. 2020;109:531–8. 10.1007/s00392-020-01626-9 PMC7087935 32161990

[14] Barron E, Bakhai C, Kar P, Weaver A, Bradley D, Ismail H, et al. Associations of type 1 and type 2 diabetes with COVID-19-related mortality in England: a whole-population study. Lancet Diabetes Endocrinol. 2020;8:813–22. 10.1016/S2213-8587(20)30272-2 PMC7426088 32798472

[15] Shi Q, Zhang X, Jiang F, Zhang X, Hu N, Bimu C, et al. Clinical characteristics and risk factors for mortality of COVID-19 patients with diabetes in Wuhan, China: a two-center, retrospective study. Diabetes Care. 2020;43:1382–91. 10.2337/dc20-0598 32409504

[16] Holman N, Knighton P, Kar P, O’Keefe J, Curley M, Weaver A, et al. Risk factors for COVID-19related mortality in people with type 1 and type 2 diabetes in England: a population-based cohort study. Lancet Diabetes Endocrinol. 2020;8:823–33. 10.1016/S2213-8587(20)30271-0 PMC7426091 32798471

[17] Guan WJ, Ni ZY, Hu Y, Liang WH, Ou CQ, He JX, et al. Clinical characteristics of coronavirus disease 2019 in China. N Engl J Med. 2020;382:1708–20. 10.1056/NEJMoa2002032 PMC7092819 32109013

[18] Wu Z, McGoogan JM. Characteristics of and important lessons from the coronavirus disease 2019 (COVID-19) outbreak in China: summary of a report of 72 314 cases from the Chinese center for disease control and prevention. J Am Med Assoc. 2020;323:1239–42. 10.1001/jama.2020.2648 32091533

[19] Guan WJ, Liang WH, Zhao Y, Liang HR, Chen ZS, Li YM, et al. Comorbidity and its impact on 1590 patients with Covid-19 in China: a nationwide analysis. Eur Respir J. 2020;55(5):2000547. 10.1183/13993003.00547-2020. PMC7098485 32217650

[20] Richardson S, Hirsch JS, Narasimhan M, Crawford JM, McGinn T, Davidson KW, et al. Presenting characteristics, comorbidities, and outcomes among 5700 patients hospitalized with COVID-19 in the New York City area. J Am Med Assoc. 2020;323:2052–9. 10.1001/jama.2020.6775 PMC7177629 32320003

[21] Chan JC, Lim LL, Wareham NJ, Shaw JE, Orchard TJ, Zhang P, et al. The lancet commission on diabetes: using data to transform diabetes care and patient lives. Lancet. 2020;396:2019–82. 10.1016/S0140-6736(20)32374-6 33189186

[22] Pearson-Stuttard J, Blundell S, Harris T, Cook DG, Critchley J. Diabetes and infection: assessing the association with glycaemic control in population-based studies. Lancet Diabetes Endocrinol. 2016;4:148–58. 10.1016/S2213-8587(15)00379-4 26656292

[23] McDonald HI, Nitsch D, Millett ER, Sinclair A, Thomas SL. New estimates of the burden of acute community‐acquired infections among older people with diabetes mellitus: a retrospective cohort study using linked electronic health records. Diabetic Med. 2014;31:606–14. 10.1111/dme.12384 PMC4264938 24341529

[24] Li S, Wang J, Zhang B, Li X, Liu Y. Diabetes mellitus and cause-specific mortality: a population-based study. Diabetes Metab J. 2019;43:319–41. 10.4093/dmj.2018.0060 PMC6581547 31210036

[25] Parohan M, Yaghoubi S, Seraji A, Javanbakht MH, Sarraf P, Djalali M. Risk factors for mortality in patients with Coronavirus disease 2019 (COVID-19) infection: a systematic review and meta-analysis of observational studies. Aging Male. 2020;1–9. 10.1080/13685538.2020.1774748. 32508193

[26] Onder G, Rezza G, Brusaferro S. Case-fatality rate and characteristics of patients dying in relation to COVID-19 in Italy. J Am Med Assoc. 2020;323:1775–6. 10.1001/jama.2020.4683 32203977

[27] Yang JK, Feng Y, Yuan MY, Yuan SY, Fu HJ, Wu BY, et al. Plasma glucose levels and diabetes are independent predictors for mortality and morbidity in patients with SARS. Diabetic Med. 2006;23:623–8. 10.1111/j.1464-5491.2006.01861.x 16759303

[28] Bode B, Garrett V, Messler J, McFarland R, Crowe J, Booth R, et al. Glycemic characteristics and clinical outcomes of COVID-19 patients hospitalized in the United States. J Diabetes Sci Technol. 2020;14:813–21. 10.1177/1932296820924469 PMC7673150 32389027

[29] Zhu L, She ZG, Cheng X, Qin JJ, Zhang XJ, Cai J, et al. Association of blood glucose control and outcomes in patients with COVID-19 and pre-existing type 2 diabetes. Cell Metab. 2020;31:1068–77. 10.1016/j.cmet.2020.04.021 PMC7252168 32369736

[30] Al Hayek AA, Robert AA, Matar AB, Algarni A, Alkubedan H, Alharbi T, et al. Risk factors for hospital admission among COVID-19 patients with diabetes. Saudi M J. 2020;41:1090–7. 10.15537/smj.2020.10.25419 PMC7841525 33026050

[31] Zhu Z, Mao Y, Chen G. Predictive value of HbA1C for adverse prognosis in COVID-19: a systematic review and meta-analysis. SSRN. 2021. ID:ppcovidwho-6421. 10.1016/j.pcd.2021.07.013 PMC8354788 34420899

[32] Sur J, Sharma J, Sharma D. Diabetes might augment the severity of COVID-19: a current prospects. Front Cardiovasc Med. 2020;7:613255. 10.3389/fcvm.2020.613255 PMC7813814 33469551

[33] Roca-Ho H, Riera M, Palau V, Pascual J, Soler MJ. Characterization of ACE and ACE2 expression within different organs of the NOD mouse. Int J Mol Sci. 2017;18:563. 10.3390/ijms18030563 PMC5372579 28273875

[34] Wysocki J, Ye M, Soler MJ, Gurley SB, Xiao HD, Bernstein KE, et al. ACE and ACE2 activity in diabetic mice. Diabetes. 2006;55:2132–9. 10.2337/db06-0033 16804085

[35] Rao S, Lau A, So HC. Exploring diseases/traits and blood proteins causally related to expression of ACE2, the putative receptor of SARS-CoV-2: a Mendelian randomization analysis highlights tentative relevance of diabetes-related traits. Diabetes Care. 2020;43:1416–26. 10.2337/dc20-0643 32430459

[36] Fernandez C, Rysä J, Almgren P, Nilsson J, Engström G, Orho‐Melander M, et al. Plasma levels of the proprotein convertase furin and incidence of diabetes and mortality. J Inter Med. 2018;284:377–87. 10.1111/joim.12783 PMC6175079 29888466

[37] Chen X, Hu W, Ling J, Mo P, Zhang Y, Jiang Q, et al. Hypertension and diabetes delay the viral clearance in COVID-19 patients. MedRxiv. 2020. 10.1101/2020.03.22.20040774.

[38] Wösten-van Asperen RM, Bos AP, Bem RA, Dierdorp BS, Dekker T, et al. Imbalance between pulmonary angiotensin-converting enzyme and angiotensin-converting enzyme 2 activity in acute respiratory distress syndrome. Pediatr Crit Care Med. 2013;14:e438–41. 10.1097/PCC.0b013e3182a55735 24226567

[39] Kuba K, Imai Y, Rao S, Gao H, Guo F, Guan B, et al. A crucial role of angiotensin converting enzyme 2 (ACE2) in SARS coronavirus – induced lung injury. Nat Med. 2005;11:875–9. 10.1038/nm1267 PMC7095783 16007097

[40] Hodgson K, Morris J, Bridson T, Govan B, Rush C, Ketheesan N. Immunological mechanisms contributing to the double burden of diabetes and intracellular bacterial infections. Immunology. 2015;144:171–85. 10.1111/imm.12394 PMC4298412 25262977

[41] Kulcsar KA, Coleman CM, Beck SE, Frieman MB. Comorbid diabetes results in immune dysregulation and enhanced disease severity following MERS-CoV infection. JCI Insight. 2019;4:e131774. 10.1172/jci.insight.131774 PMC6824443 31550243

[42] Xu Z, Shi L, Wang Y, Zhang J, Huang L, Zhang C, et al. Pathological findings of COVID-19 associated with acute respiratory distress syndrome. Lancet Respir Med. 2020;8:420–2. 10.1016/S2213-2600(20)30076-X PMC7164771 32085846

[43] Yang X, Yu Y, Xu J, Shu H, Liu H, Wu Y, et al. Clinical course and outcomes of critically ill patients with SARS-CoV-2 pneumonia in Wuhan, China: a single-centered, retrospective, observational study. Lancet. Respir Med. 2020;8:475–81. 10.1016/S2213-2600(20)30079-5 PMC7102538 32105632

[44] Apicella M, Campopiano MC, Mantuano M, Mazoni L, Coppelli A, Del Prato S. COVID-19 in people with diabetes: understanding the reasons for worse outcomes. Lancet Diabetes Endocrinol. 2020;8:782–92. 10.1016/S2213-8587(20)30238-2 PMC7367664 32687793

[45] Critchley JA, Carey IM, Harris T, DeWilde S, Hosking FJ, et al. Glycemic control and risk of infections among people with type 1 or type 2 diabetes in a large primary care cohort study. Diabetes Care. 2018;41:2127–35. 10.2337/dc18-0287 30104296

[46] Muniyappa R, Gubbi S. COVID-19 pandemic, coronaviruses, and diabetes mellitus. Am J Physiol Endocrinol Metab. 2020;318:E736–41. 10.1152/ajpendo.00124.2020 PMC7191633 32228322

[47] Jafar N, Edriss H, Nugent K. The effect of short-term hyperglycemia on the innate immune system. Am J Med Sci. 2016;351:201–11. 10.1016/j.amjms.2015.11.011 26897277

[48] Wang Q, Fang P, He R, Li M, Yu H, Zhou L, et al. O-GlcNAc transferase promotes influenza A virus–induced cytokine storm by targeting interferon regulatory factor–5. Sci Adv. 2020;6:eaaz7086. 10.1126/sciadv.aaz7086. PMC7159909 32494619

[49] Bornstein SR, Rubino F, Khunti K, Mingrone G, Hopkins D, Birkenfeld AL, et al. Practical recommendations for the management of diabetes in patients with COVID-19. Lancet Diabetes Endocrinol. 2020;8:546–50. 10.1016/S2213-8587(20)30152-2 PMC7180013 32334646

[50] Yang JK, Lin SS, Ji XJ, Guo LM, Ji X, Ji X, et al. Binding of SARS coronavirus to its receptor damages islets and causes acute diabetes. Acta Diabetol. 2010;47:193–9. 10.1007/s00592-009-0109-4 PMC7088164 19333547

[51] Varga Z, Flammer AJ, Steiger P, Haberecker M, Andermatt R, Zinkernagel AS, et al. Endothelial cell infection and endotheliitis in COVID-19. Lancet. 2020;395(10234):1417–8. 10.1016/S0140-6736(20)30937-5 PMC7172722 32325026

[52] Moutschen MP, Scheen AJ, Lefebvre PJ. Impaired immune responses in diabetes mellitus: analysis of the factors and mechanisms involved. Relevance to the increased susceptibility of diabetic patients to specific infections. Diabete Metab. 1992;18:187–201. 1397473

[53] Schuetz P, Castro P, Shapiro NI. Diabetes and sepsis: preclinical findings and clinical relevance. Diabetes Care. 2011;34:771–89. 10.2337/dc10-1185 PMC3041224 21357364

[54] Tariq S, Tariq S, Baig M, Saeed M. Knowledge, awareness and practices regarding novel coronavirus among a sample of Pakistani population, a cross-sectional study. Disaster Med Public Health Prep. 2020;1–20. 10.1017/dmp.2020.408. PMC7943951 33092681

[55] Kumar A, Arora A, Sharma P, Anikhindi SA, Bansal N, Singla V, et al. Is diabetes mellitus associated with mortality and severity of COVID-19? A meta-analysis. Diabetes Metab Syndr Clin Res Rev. 2020;14:535–45. 10.1016/j.dsx.2020.04.044 PMC7200339 32408118

[56] Wei Y-Y, Wang R-R, Zhang D-W, Tu Y-H, Chen C-S, Ji S, et al. Risk factors for severe COVID-19: evidence from 167 hospitalized patients in Anhui, China. J Infect. 2020;81:e89–92. 10.1016/j.jinf.2020.04.010 PMC7162743 32305487

[57] Simonnet A, Chetboun M, Poissy J, Raverdy V, Noulette J, Duhamel A, et al. High prevalence of obesity in severe acute respiratory syndrome coronavirus‐2 (SARS‐CoV‐2) requiring invasive mechanical ventilation. Obesity. 2020;28:1195–9. 10.1002/oby.22831 PMC7262326 32271993

[58] Wu C, Chen X, Cai Y, Xia J, Zhou X, Xu S, et al. Risk factors associated with acute respiratory distress syndrome and death in patients with coronavirus disease 2019 pneumonia in Wuhan. China JAMA Intern Med. 2020;180:934–43. 10.1001/jamainternmed.2020.0994 PMC7070509 32167524

[59] Mo P, Xing Y, Xiao Y, Deng L, Zhao Q, Wang H, et al. Clinical characteristics of refractory COVID-19 pneumonia in Wuhan, China. Clin Infect Dis. 2020. 10.1093/cid/ciaa270. PMC7184444 32173725

[60] Huang I, Lim MA, Pranata R. Diabetes mellitus is associated with increased mortality and severity of disease in COVID‐19 pneumonia a systematic review, meta‐analysis, and meta‐regression. Diabetes Metab Syndr. 2020;14:395–403. 10.1016/j.dsx.2020.04.018 PMC7162793 32334395

[61] Tian W, Jiang W, Yao J, Nicholson CJ, Li RH, Sigurslid HH, et al. Predictors of mortality in hospitalized COVID‐19 patients: a systematic review and meta‐analysis. J Med Virol. 2020;92:1875–83. 10.1002/jmv.26050 PMC7280666 32441789

